# Effects of hemoglobin transfusion thresholds on clinical outcomes in sepsis-related ARDS: A retrospective cohort analysis utilizing the MIMIC-IV database

**DOI:** 10.1097/MD.0000000000044790

**Published:** 2025-09-26

**Authors:** Kexin Wen, Tianyu Zhao, Ruimin Tan, Xumin Han, Lei Li, Quansheng Du

**Affiliations:** a North China University of Science and Technology, Tangshan City, Hebei Province, China; b Department of Intensive Care Unit, Hebei Provincial People’s Hospital, Shijiazhuang City, Hebei Province, China; c Hebei North University, Zhangjiakou City, Hebei Province, China; d Hebei Medical University, Shijiazhuang City, Hebei Province China.

**Keywords:** acute respiratory distress syndrome, conservative transfusion strategy, sepsis, transfusion thresholds

## Abstract

Sepsis, a systemic pathological reaction induced by infection, is a major contributor to acute respiratory distress syndrome (ARDS) in intensive care units (ICUs). This retrospective cohort study aimed to evaluate the impact of hemoglobin transfusion thresholds on clinical outcomes in patients with sepsis-related ARDS using the medical information mart for intensive care IV database. Patients were categorized into a high-threshold group (hemoglobin ≥ 7 g/dL) and a low-threshold group (hemoglobin < 7 g/dL) based on the lowest hemoglobin concentration within 24 hours before transfusion. The results showed no significant differences in overall mortality during hospitalization, in the ICU, or at 30, 60, and 90 days between the high- and low-transfusion threshold groups. The duration of ICU stay was comparable in both groups; however, the total hospital stay was longer in the low-threshold cohort. Stratified analysis showed that changing the transfusion thresholds did not significantly affect the prognosis of people with ARDS stage 1. However, in ARDS stage 2, the high-threshold group showed a trend toward lower in-hospital and ICU mortality rates, although the difference was not statistically significant. This study suggests that a low-transfusion threshold does not adversely affect sepsis-related ARDS patient mortality, but the potential benefits of blood transfusion need to be balanced against the risk of prolonged hospitalization.

## 1. Introduction

Sepsis is a systemic inflammatory response syndrome triggered by infection that is characterized by increased incidence and mortality rates and has become a major global public health concern.^[1–3]^ Although medical technology has progressed, resulting in decreased mortality rates for sepsis patients, it continues to be a predominant cause of death in intensive care unit (ICU) patients and is likely among the diseases responsible for the highest global mortality rates.^[4,5]^ Studies indicate that the fatality rate of sepsis is approximately 25% to 45%,^[6]^ and the impact of sepsis-related complications on patient prognosis cannot be overlooked,as acute respiratory distress syndrome (ARDS) is the most prevalent complication of sepsis.^[7]^ Sepsis-induced acute respiratory distress syndrome (S-ARDS) is characterized by the rapid onset of non-cardiogenic pulmonary edema, diffuse bilateral infiltrates, and severe hypoxemia. Its pathogenesis involves excessive neutrophil activation, release of inflammatory mediators, oxidative stress, and fibroproliferation, which disrupt the alveolar-capillary barrier and impair gas exchange, ultimately leading to respiratory failure and high mortality.^[8,9]^ Clinically, patients typically present with dyspnea, refractory hypoxemia, and radiographic evidence of diffuse pulmonary infiltrates. The systemic inflammatory response associated with sepsis further exacerbates pulmonary microcirculatory dysfunction and alveolar epithelial injury, thereby aggravating pulmonary edema and oxygenation impairment.^[10,11]^ The key to managing S-ARDS is its early recognition and intervention. Timely control of the infection source, optimization of hemodynamics, fluid resuscitation, and mechanical ventilation support are the main strategies currently employed in the management of S-ARDS. Research has demonstrated that early blood transfusion can improve systemic oxygen delivery in patients with sepsis, thereby helping to compensate for impaired pulmonary gas exchange. In contrast, while fluid resuscitation can improve hemodynamic status, it has not been shown to effectively enhance pulmonary microcirculation function.

For patients with sepsis, the choice of the transfusion strategy requires careful consideration. Despite the prevalent use of blood transfusions to address anemia and improve tissue oxygenation, a definitive consensus on the ideal transfusion threshold remains elusive. Current research suggests a minimal disparity in death and ischemia event rates among sepsis patients with elevated versus reduced transfusion thresholds. The Surviving Sepsis Campaign states that a hemoglobin level of 7 g/dL is the minimum level needed for transfusion, unless there is acute cardiac ischemia, severe hypoxemia, or sudden bleeding.^[12]^ Nevertheless, transfusion decisions for patients with S-ARDS should comprehensively consider hemodynamic stability, etiology of sepsis, complications, and overall clinical condition. Numerous guidelines endorse a limited transfusion strategy for critically ill patients, particularly for those with sepsis. Researchers have determined that a restrictive transfusion approach (hemoglobin < 7.5 g/dL) can reduce the necessity for red blood cell transfusions without increasing the risk of acute kidney injury in individuals who have undergone cardiac surgery. This study seeks to examine the effects of varying transfusion thresholds (≥7 vs <7 g/dL) on patient outcomes by a retrospective review of clinical data from individuals with sepsis-induced acute lung damage. It is hoped that this study will provide further clinical evidence for transfusion strategies and optimize treatment plans, thereby enhancing patient outcomes.

## 2. Materials and methods

### 2.1. Data source

The data for this study were obtained from the publicly available medical information mart for intensive care IV (MIMIC-IV3.1) database, which was collaboratively developed and administered by the Beth Israel Deaconess Medical Center (BIDMC) and Massachusetts Institute of Technology. The MIMIC-IV database is available to healthcare professionals globally and comprises comprehensive hospitalization information for 55,369 patients and 76,943 ICU admissions at the BIDMC from 2008 to 2019. The database contains demographic data, vital signs, medical orders, diagnoses, laboratory tests, and outcomes. All data were anonymized to safeguard patient confidentiality. The establishment of the database was sanctioned by the Institutional Review Boards of Massachusetts Institute of Technology and BIDMC, and because the data were de-identified, informed consent from patients was not required for this study. The MIMIC-IV database is a publicly accessible resource from which data were retrieved by one of the authors, who completed the online training course on data or specimens only in human research (certification number: 14,292,928).

### 2.2. Study population

This study included all adult patients (aged ≥ 18 years) who fulfilled the diagnostic criteria for sepsis as delineated by the Third International Consensus Definitions for Sepsis and Septic Shock (Sepsis-3.0). The diagnosis of ARDS was based on the symptoms, imaging results, and arterial blood gas analysis. ARDS is usually sudden, has bilateral pulmonary infiltrates, has a PaO_2_/FiO_2_ ratio ≤ 300 mm Hg, and cannot be fully explained by heart failure or fluid overload. Mild (ARDS stage 1): positive end-expiratory pressure/continuous positive airway pressure (PEEP/CPAP) ≥ 5 cm H_2_O, 200 mm Hg < PaO_2_/FiO_2_ ≤ 300 mm Hg; moderate (ARDS stage 2): PEEP/CPAP ≥ 5 cm H_2_O, 100 mm Hg < PaO_2_/FiO_2_ ≤ 200 mm Hg; severe (ARDS stage 3): PEEP/CPAP ≥ 5 cm H_2_O, PaO_2_/FiO_2_ ≤ 100 mm Hg. All patients were admitted to the ICU for the first time, with a hospital stay of ≥1 d, and received blood transfusions. Patients were divided into a high-threshold group (hemoglobin ≥ 7 g/dL) and a low-threshold group (hemoglobin < 7 g/dL) based on the minimum hemoglobin value within 24 hours prior to transfusion (Figure S1, Supplemental Digital Content, https://links.lww.com/MD/Q144).

Exclusion criteria included:

1. Multiple hospital admissions, with only the first admission retained;
2. ICU stay < 1 day;
3. Patients who were not receiving blood transfusions;
4. Patients with incomplete data.

### 2.3. Study data

This study extracted extensive clinical data from the database using Structured Query Language for data retrieval. Specifically, the extracted data included the following:

1. Demographic parameters: sex, age, and race (White, Black, or other);
2. Comorbidities: Myocardial infarction, congestive heart failure, cerebrovascular disease, chronic pulmonary disease, diabetes, liver disease, and renal disease;
3. Vital signs: heart rate, mean arterial pressure, respiratory rate, and oxygen saturation;
4. The following laboratory tests were used to check for problems: hemoglobin, platelets, white blood cells, international normalized ratio, prothrombin time, activated partial thromboplastin time, creatinine, potassium, sodium, bicarbonate, lactate, PaO_2_, PaCO_2_, and pH;
5. Other parameters: Sequential organ failure assessment (SOFA) score.

The principal endpoint of the study was to compare notable disparities in in-hospital mortality and ICU mortality between the 2 cohorts. Secondary outcomes included evaluation of in-hospital mortality and ICU mortality at 30, 60, and 90 days, along with notable discrepancies in the ICU and hospital length of stay.

### 2.4. Statistical analysis

Continuous variables with a normal distribution are presented as mean ± standard deviation, whereas non-normally distributed continuous variables are represented as median and interquartile range (IQR). Categorical variables were examined using the chi-square test, normally distributed continuous variables were assessed using one-way analysis of variance, non-normally distributed continuous variables were evaluated with the Kruskal–Wallis test, and Cox regression was used to analyze the association between the 2 groups and the primary result.

The primary outcome analysis was conducted after imputing the missing data using the random forest method to mitigate bias. Propensity score matching (PSM) was performed using all baseline variables presented in Table 1, including demographic characteristics, ARDS stage, SOFA score, vital signs, comorbidities, and laboratory values. A standardized mean difference of <0.1 indicates a fair comparison between the groups.

**Table 1:** Baseline characteristics of patients.

Variable	Pre-matched cohort				Post-matched cohort			
	High-threshold group	Low-threshold group	*P*-value	SMD	High-threshold group	Low-threshold group	*P*-value	SMD
N	5077	1134			1134	1134		
General information								
Age (yr)	64.45 ± 14.83	62.81 ± 16.02	.001	0.106	62.47 ± 15.09	62.81 ± 16.02	.600	0.022
Male (%)	3073 (60.5)	583 (51.4)	<.001	0.184	572 (50.4)	583 (51.4)	.674	0.019
Race (%)			<.001	0.143			.802	0.028
White	3378 (66.5)	692 (61.0)			682 (60.1)	692 (61.0)		
Black	375 (7.4)	125 (11.0)			121 (10.7)	125 (11.0)		
Other	1324 (26.1)	317 (28.0)			331 (29.2)	317 (28.0)		
ARDS stage (%)			<.001	0.192			.555	0.046
Stage 1	1779 (35.0)	484 (42.7)			491 (43.3)	484 (42.7)		
Stage 2	1932 (38.1)	425 (37.5)			438 (38.6)	425 (37.5)		
Stage 3	1366 (26.9)	225 (19.8)			205 (18.1)	225 (19.8)		
SOFA score vital signs	8.33 ± 3.88	10.29 ± 4.08	<.001	0.491	10.30 ± 4.15	10.29 ± 4.08	.947	0.003
Heart rate (beats/min)	72.68 ± 15.61	73.81 ± 15.81	.029	0.072	74.12 ± 17.31	73.81 ± 15.81	.649	0.019
Respiratory rate (breaths/min)	11.98 ± 4.13	12.24 ± 4.10	.053	0.064	12.26 ± 4.36	12.24 ± 4.10	.889	0.006
Mean arterial pressure (mm Hg)	53.85 ± 12.94	52.68 ± 13.42	.007	0.088	52.59 ± 14.11	52.68 ± 13.42	.875	0.007
Oxygen saturation (%)	90.89 ± 8.68	90.02 ± 10.69	.003	0.089	90.10 ± 10.60	90.02 ± 10.69	.859	0.007
Comorbidities (%)								
Myocardial infarction	1152 (22.7)	211 (18.6)	.003	0.101	204 (18.0)	211 (18.6)	.745	0.016
Congestive heart failure	688 (13.6)	164 (14.5)	.448	0.026	357 (31.5)	373 (32.9)	.500	0.03
Cerebrovascular disease	1387 (27.3)	294 (25.9)	.053	0.032	168 (14.8)	164 (14.5)	.859	0.010
Liver disease	1105 (21.8)	347 (30.6)	<.001	0.202	361 (31.8)	347 (30.6)	.556	0.027
Diabetes	1619 (31.9)	330 (29.1)	.073	0.061	328 (28.9)	330 (29.1)	.963	0.004
Renal disease	1184 (23.3)	317 (28.0)	.001	0.106	308 (27.2)	317 (28.0)	.707	0.018
Malignancy	1278(25.17)	331(29.1)	<.001	0.160	294(25.9)	304(26.8)	.964	0.003
Laboratory tests								
White blood cells (×10^9^/L)	10.96 ± 8.39	9.73 ± 7.04	<.001	0.158	9.79 ± 6.67	9.73 ± 7.04	.841	0.008
Platelets (×10^9^/L)	151.76 ± 101.26	124.66 ± 117.40	<.001	0.247	188.80 ± 131.08	186.61 ± 140.39	.639	0.015
International normalized ratio (INR)	1.39 ± 0.56	1.47 ± 0.53	<.001	0.143	1.45 ± 0.56	1.47 ± 0.53	.410	0.035
Prothrombin time (s)	15.33 ± 5.81	16.07 ± 5.70	<.001	0.129	15.86 ± 5.76	16.07 ± 5.70	.378	0.037
Activated partial thromboplastin time (s)	32.49 ± 10.31	32.99 ± 11.14	.143	0.047	33.06 ± 9.78	32.99 ± 11.14	.887	0.006
Bicarbonate (mmol/L)	20.22 ± 4.83	18.12 ± 5.59	<.001	0.403	18.20 ± 5.25	18.12 ± 5.59	.721	0.015
Potassium (mmol/L)	3.96 ± 0.58	3.95 ± 0.65	.443	0.024	3.93 ± 0.63	3.95 ± 0.65	.458	0.031
Sodium (mmol/L)	136.72 ± 4.91	136.77 ± 5.51	.772	0.009	136.68 ± 5.54	136.77 ± 5.51	.687	0.017
Creatinine (mg/dL)	1.41 ± 1.41	1.68 ± 1.53	<.001	0.186	1.67 ± 1.57	1.68 ± 1.53	.889	0.006
pH	7.26 ± 0.11	7.22 ± 0.14	<.001	0.317	7.22 ± 0.13	7.22 ± 0.14	.861	0.007
Partial pressure of oxygen (mm Hg)	73.09 ± 32.99	61.49 ± 31.74	<.001	0.359	61.28 ± 27.78	61.49 ± 31.74	.870	0.007
Partial pressure of carbon dioxide (mm Hg)	33.92 ± 7.15	32.97 ± 7.93	<.001	0.127	32.9 ± 7.64	32.97 ± 7.93	.948	0.003
Lactate (mmol/L)	1.76 ± 1.38	2.18 ± 2.07	<.001	0.238	2.14 ± 1.93	2.18 ± 2.07	.670	0.018

ARDS = acute respiratory distress syndrome, SMD = standardized mean difference.

The Kaplan–Meier method and Cox regression were utilized in the matched cohort to analyze in-hospital mortality, ICU mortality, and mortality rates at 30, 60, and 90 days. The Wilcoxon rank-sum test was used to compare the average length of stay in the intensive care unit and hospital between the 2 groups. Subgroup analyses were performed to evaluate variations in in-hospital mortality among different categories. In addition, hemoglobin concentration was further analyzed as a continuous variable using restricted cubic spline (RCS) regression to explore potential nonlinear associations with clinical outcomes.

All statistical analyses were performed using R software version 4.2.2 (R Foundation for Statistical Computing, Vienna, Austria), and statistical significance was established at a 2-sided *P*-value of <.05.

## 3. Results

### 3.1. Baseline characteristics of patients

Based on the analysis of the table data, the pre-matched cohort included 6211 patients, with 5077 in the high-threshold group and 1134 in the low-threshold group. Among these patients, 36.44% had ARDS stage 1, 37.95% had ARDS stage 2, and 25.62% had ARDS stage 3. Compared to the high-threshold group, the low-threshold group exhibited a reduced incidence of myocardial infarction (18.6%) but an elevated incidence of acute heart failure (14.5%) and liver disease (30.6%).

After PSM, 1,134 patients from the high-threshold group and 1,134 patients from the low-threshold group were successfully matched, thereby reducing the interference of confounding factors. In total, 2268 patients were included in the analysis. Table 1 presents the baseline characteristics of the 2 groups before and after PSM, including sex, age, race, SOFA score, vital signs, underlying diseases, and laboratory test outcomes.

Prior to matching, patients in the high-threshold group were older, had fewer females, and had a higher proportion of ARDS stage 3 ARDS than those in the low-threshold group. It was more common for people in the low-threshold group to have congestive heart failure, liver disease, or kidney disease. Their vital signs were less stable, with a faster heart rate, tachypnea, lower mean arterial pressure, and lower oxygen saturation. They also had worse coagulation function, with a higher international normalized ratio, longer prothrombin time, and longer activated partial thromboplastin time. Additionally, they exhibit higher creatinine levels and worse microcirculatory perfusion (higher lactate levels). After PSM, the baseline parameters of the low- and high-threshold categories were equilibrated, with a comparable number of patients in each group (1134 in each group), thus ensuring the comparability of subsequent studies.

### 3.2. Hemoglobin concentration and transfusion thresholds

**Figure 1.**
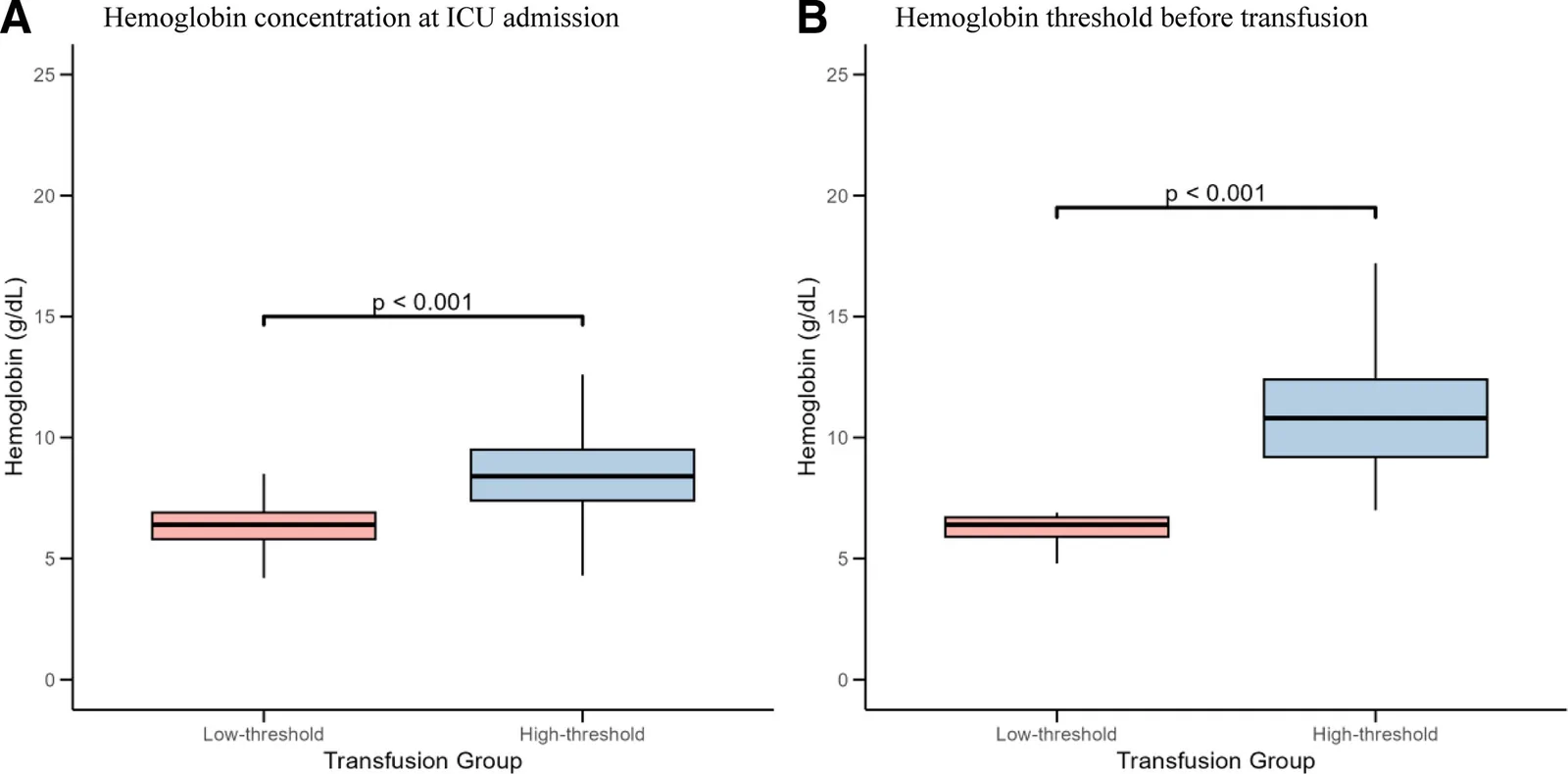
Hemoglobin concentrations in the 2 groups: (A) Hemoglobin concentration at ICU admission. (B) Hemoglobin threshold before transfusion. ICUs = intensive care units.

**Figure 2.**
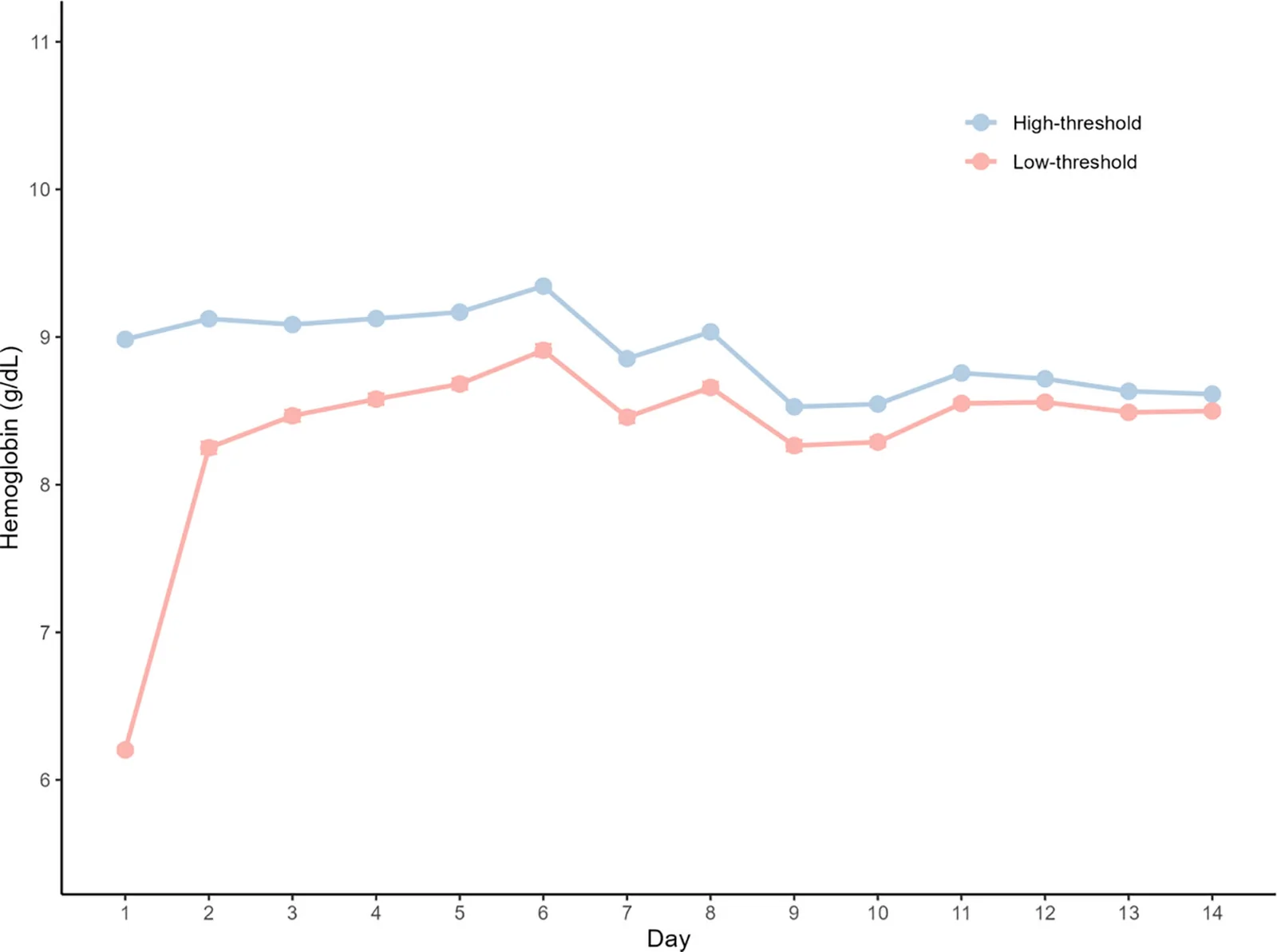
Comparison of hemoglobin concentration trends at ICU admission and within 14 days posttransfusion. ICUs = intensive care units.

In the matched cohort, the low-threshold group exhibited a transfusion threshold of 6.4 g/dL (IQR: 6.0–6.7), while the high-threshold group demonstrated a transfusion threshold of 8.7 g/dL (IQR: 7.8–9.8) (*P* < .001) (Fig. 1B). Furthermore, hemoglobin concentrations within the initial 24 hours post-ICU admission were compared between the 2 cohorts. The results showed a significant difference between the high-threshold group (11 g/dL [IQR, 9.4–12.6]) and low-threshold group (8.5 g/dL [IQR, 7.1–10.6]) (*P* < .001) (Fig. 1A). In addition, the trend of changes in hemoglobin concentration from admission to the intensive care unit to 14 days after transfusion (Fig. 2) showed that there was a significant increase in hemoglobin concentration on the first day after transfusion (*P* < .001), which then gradually leveled off over the next few days.

### 3.3. Analysis of outcomes

#### 3.3.1. Primary outcomes

**Figure 3.**
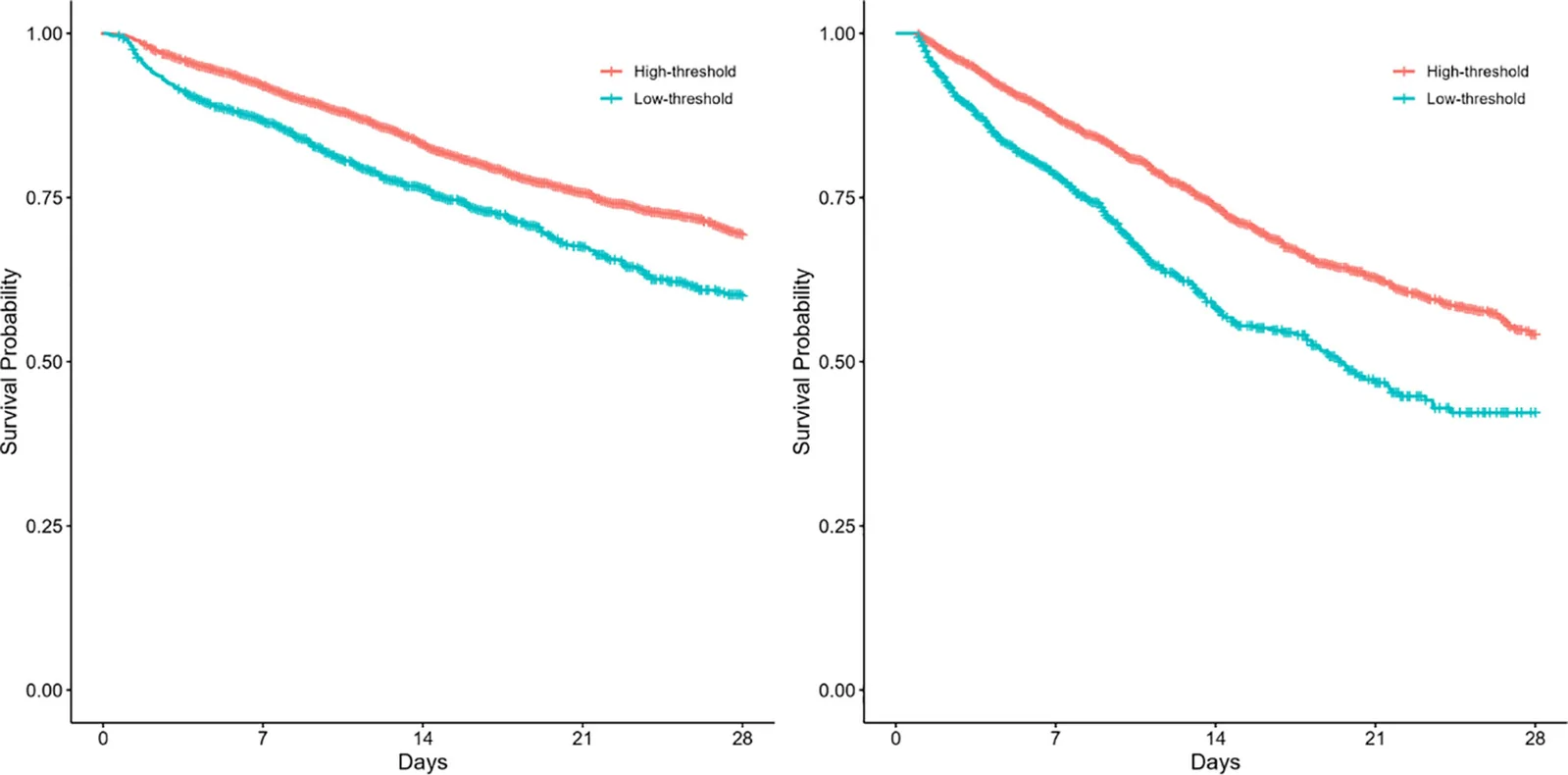
(A) Kaplan–Meier curve for in-hospital survival probability. (B) Kaplan–Meier curve for ICU survival probability. ICUs = intensive care units.

**Figure 4.**
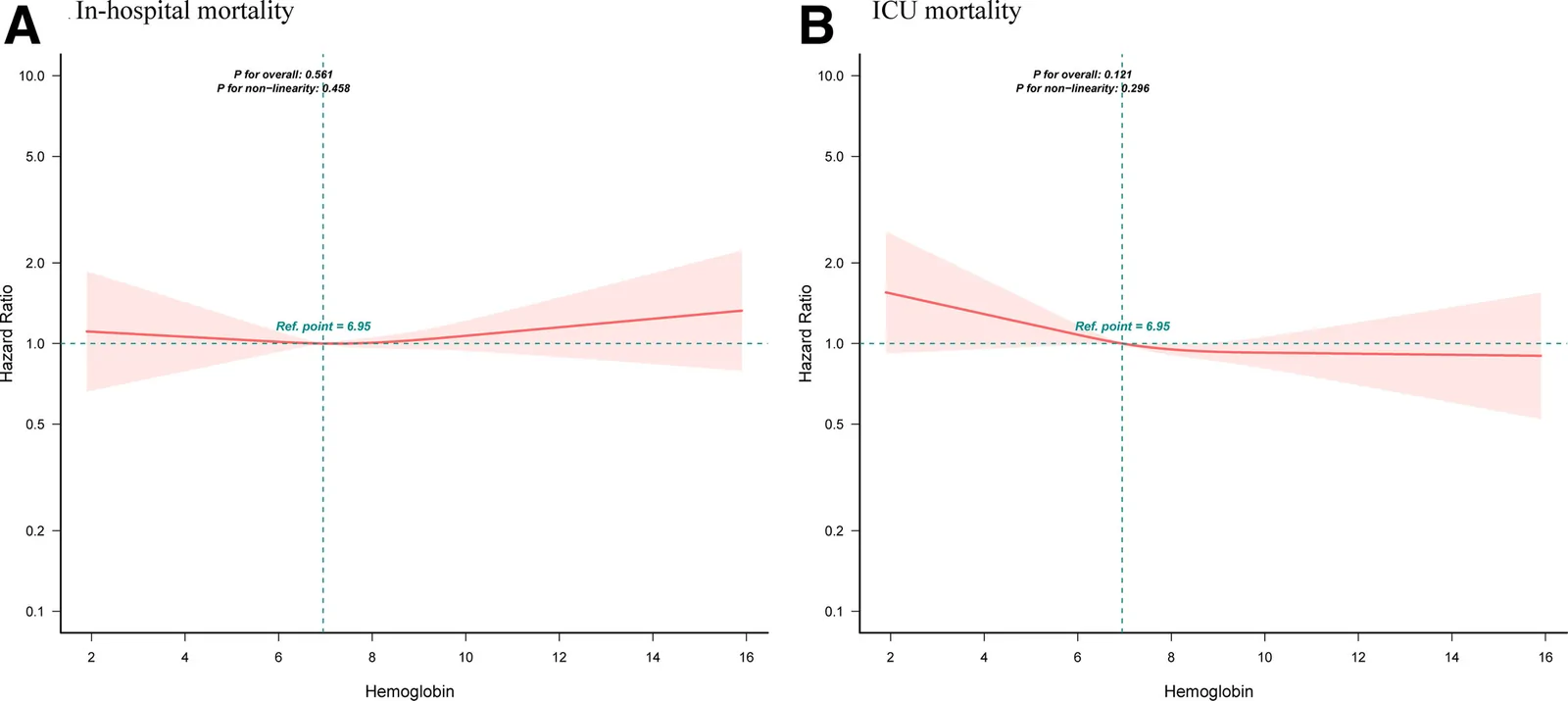
RCS analysis of hemoglobin threshold before transfusion and mortality: (A) In-hospital mortality. (B) ICU mortality. ICUs = intensive care units, RCS = restricted cubic spline.

In the matched cohort, the in-hospital mortality rate was 29.28% (340/1134) in the high-threshold group and 34.83% (395/1134) in the low-threshold group. The ICU mortality rate was 25.57% (290/1134) in the high-threshold group and 29.45% (334/1134) in the low-threshold group (Table 2). Kaplan–Meier survival analysis and Cox regression analysis showed that there were no significant differences between the high-threshold and low-threshold groups in the number of deaths in the hospital or ICU (hazard ratio [HR]: 1.06; 95% confidence interval [CI]: 0.91–1.23; *P* = .44; HR: 1.17; 95% CI: 0.99–1.37; *P* = .07) (Fig. 3). To further address the limitation of dichotomizing hemoglobin thresholds, we analyzed hemoglobin concentration as a continuous variable. As shown in Table 3, baseline hemoglobin levels were not significantly associated with in-hospital mortality (HR: 1.02, 95% CI: 0.98–1.06; *P* = .446) or ICU mortality (HR: 0.96, 95% CI: 0.93–1.01; *P* = .087). RCS models likewise revealed no significant nonlinear associations (Fig. 4), with curves showing a flat risk profile across the clinical range, suggesting no clear threshold effect.

**Table 2:** Comparison of outcomes between high-threshold and low-threshold groups.

Outcome	High-threshold group (n = 1134)	Low-threshold group (n = 1134)	HR or average difference (95% CI)	*P*-value
Primary outcomes				
In-hospital mortality (%)	340 (29.28)	395 (34.83)	1.06 (0.91, 1.23)	.44
ICU mortality (%)	290 (25.57)	334 (29.45)	1.17 (0.99, 1.37)	.07
Secondary outcomes				
30-d mortality (%)	327 (28.8)	396 (34.9)	1.13 (0.97, 1.31)	.12
60-d mortality (%)	378 (33)	468 (41.2)	1.15 (1, 1.32)	.05
90-d mortality (%)	398 (35.1)	491 (43.3)	1.15 (1, 1.31)	.05
ICU length of stay (d)	9.18 (3.4–13.0)	8.62 (3.3–13.6)	‐0.30 (‐1.2, 0.7)	.58
Hospital length of stay (d)	14.2 (8.3–24.0)	16.0 (8.9–27.0)	‐2.18 (‐3.49, ‐0.88)	.114

CI = confidence interval, HR = hazard ratio, ICUs = intensive care units.

**Table 3:** The association between continuous hemoglobin levels before transfusion and primary outcomes.

Outcome	Survival	Dead	HR (95% CI)	*P*-value
In-hospital mortality	7.58 ± 1.79	7.61 ± 1.97	1.02 (0.98–1.06)	.446
ICU mortality	7.60 ± 1.82	7.56 ± 1.89	0.96 (0.93–1.01)	.087

CI = confidence interval, ICUs = intensive care units.

#### 3.3.2. Secondary outcomes

**Figure 5.**
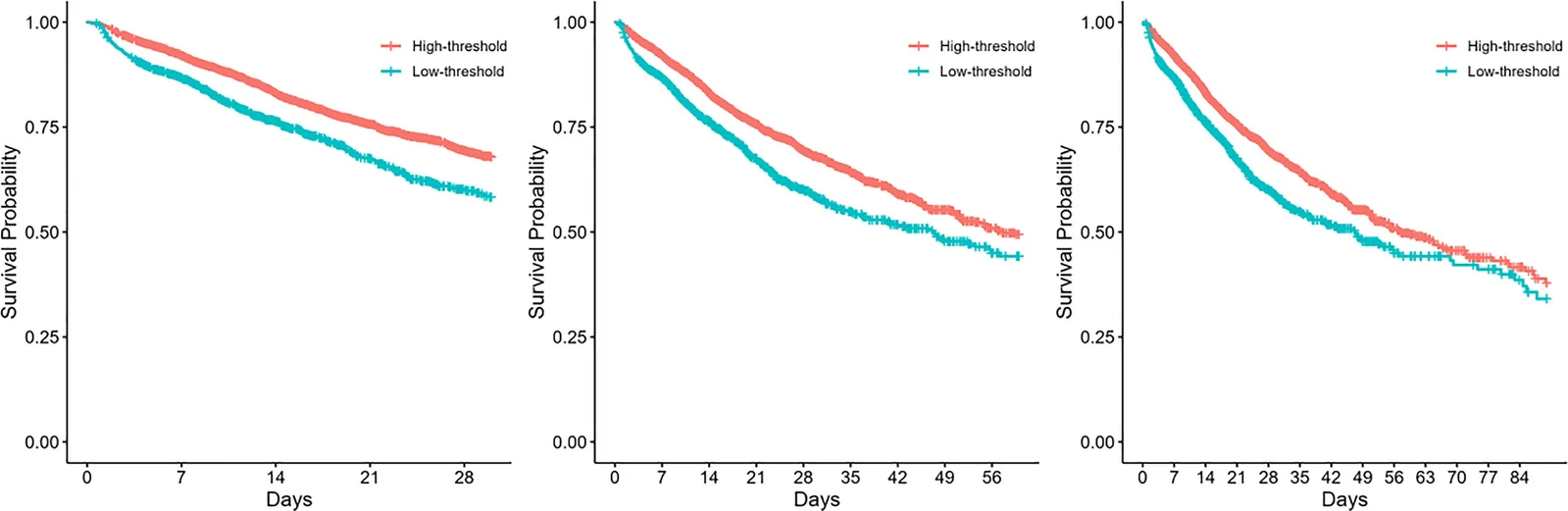
(A) Kaplan–Meier curve for 30-day survival probability. (B) Kaplan–Meier curve for 60-day survival probability. (C) Kaplan–Meier curve for 90-day survival.

No substantial differences were observed in the 30-day, 60-day, and 90-day mortality rates between the high- and low-threshold groups (HR: 1.13; 95% CI: 0.97–1.31; *P* = .12; HR: 1.15; 95% CI: 1–1.32; *P* = .05; HR: 1.15; 95% CI: 1–1.31; *P* = .05) (Fig. 5). In the comparison of hospital and ICU length of stay, no significant difference was observed in ICU length of stay between the 2 groups (9.18 vs 8.26 days, *P* = .58). Additionally, the overall length of hospital stay was not significantly longer in the low-threshold group than in the high-threshold group (14.2 vs 16 days, *P* < .114) (Table 2).

### 3.4. Analysis of the relationship between ARDS stages and mortality

The differences in in-hospital and ICU mortality among patients with different ARDS stages are presented in Table 4. For ARDS stage 1 patients, no significant differences were observed between the high- and low-threshold groups, indicating that transfusion thresholds had a minimal impact on outcomes. The high-threshold group had lower in-hospital and ICU mortality rates for ARDS stage 2 ARDS, although the differences were not statistically significant. This finding suggests that higher transfusion thresholds may have a protective effect. However, for ARDS stage 3 ARDS, the ICU death rate was markedly elevated in the high-threshold group, suggesting that overly elevated transfusion thresholds may negatively affect the prognosis of critically ill patients. Therefore, transfusion strategies should be adjusted based on disease severity in clinical practice to avoid overintervention.

**Table 4:** Relationship between ARDS stages and in-hospital mortality and ICU mortality.

Outcome	High-threshold group	Low-threshold group	HR (95% CI)	*P*-value
ARDS stage 1	1779	484		
In-hospital mortality (%)	566 (31.82)	211 (43.6)	1.001 (0.85–1.19)	.993
ICU mortality (%)	496 (27.88)	104 (21.48)	1.18 (0.825–1.687)	.365
ARDS stage 2	1931	425		
In-hospital mortality (%)	371 (19.21)	132 (31.06)	1.117 (0.9–1.386)	.314
ICU mortality (%)	317 (16.42)	111 (26.12)	1.314 (1.034–1.669)	.025
ARDS stage 3	1366	225		
In-hospital mortality (%)	203 (14.86)	52 (23.11)	1.081 (0.78–1.498)	.641
ICU mortality (%)	159 (11.64)	81 (36)	1.3 (1.08–1.56)	.005

ARDS = acute respiratory distress syndrome, CI = confidence interval, ICUs = intensive care units, HR = hazard ratio.

## 4. Discussion

This study investigated the impact of different hemoglobin transfusion thresholds on outcomes in patients with S-ARDS using a retrospective cohort analysis. We found no significant differences in in-hospital or ICU mortality between patients managed with a higher threshold (≥7 g/dL) and those managed with a lower threshold (<7 g/dL). To strengthen these findings, we further analyzed hemoglobin concentration as a continuous variable. RCS modeling demonstrated significant associations between pre-transfusion hemoglobin levels and mortality, with risk curves showing a relatively flat profile across the observed clinical range. These results suggest that hemoglobin concentration does not exert a discrete threshold effect on outcomes in S-ARDS, but rather reflects a more stable risk spectrum. Accordingly, transfusion decisions should not be based solely on rigid numeric cutoffs, but should be individualized according to clinical context, including hemodynamic status, comorbid conditions, and disease severity.

Notably, despite no notable difference in ICU length of stay, the overall hospital stay was significantly extended in the low-threshold group. This finding highlights the complexity of transfusion strategies in the management of patients with S-ARDS, and underscores the importance of tailoring transfusion protocols to individual patients. The pathophysiological mechanisms of S-ARDS are complex and involve multiple factors such as inflammatory responses, microvascular dysfunction, and alveolar epithelial cell injury. Pathogen-associated molecular patterns or damage-associated molecular patterns bind to host cell receptors and trigger the release of inflammatory mediators, which leads to pulmonary microcirculatory dysfunction, inadequate tissue perfusion, and organ dysfunction.^[13]^ The activation of endothelial cells by inflammation further exacerbates leukocyte adhesion and rolling, capillary leakage, and other processes, thereby worsening lung injury.^[14]^ At the cellular level, mitochondrial dysfunction and production of reactive oxygen species during sepsis directly damage alveolar epithelial cells. For example, lipopolysaccharide binds to TLR-4 receptors, activating pro-apoptotic signals or triggering an increase in intracellular reactive oxygen species and nitrogen species, leading to mitochondrial-dependent apoptosis in alveolar epithelial cells.^[15]^ Theoretically, blood transfusions may improve lung function in patients with S-ARDS by correcting anemia, replenishing blood volume, and enhancing oxygenation. However, existing studies have shown that the effect of transfusions on mortality in critically ill patients remains controversial.

Some studies have indicated that transfusions do not significantly improve mortality rates in critically ill patients. For example, a randomized controlled trial by Hebert et al found no significant difference in 30-day mortality between liberal and restrictive transfusion strategies.^[16]^ Similarly, Odutayo et al found that restrictive transfusion strategies reduce all-cause mortality and rebleeding risk in patients with gastrointestinal bleeding.^[17]^ These studies indicate that restrictive transfusion criteria are not less effective than liberal thresholds for reducing mortality and side effects. Notably, although transfusions may positively affect lung function by improving oxygenation, their potential risks cannot be ignored. Plataki et al found that transfusions were independently associated with acute kidney injury in patients with septic shock, highlighting the potential harmful effects of transfusion.^[18]^

An important practical implication of our results is that the transfusion threshold groups were not entirely the result of discretionary clinical decision-making. Patients in the low-threshold group generally presented with hemoglobin values already below 7 g/dL at ICU admission, leaving clinicians little choice but to transfuse promptly. Thus, the only actionable policy question concerns whether transfusion in patients with higher initial hemoglobin can safely be deferred until lower thresholds are reached. Recognizing this asymmetry has 2 clinical implications. First, our results should not be interpreted as supporting permissive tolerance of progressively lower hemoglobin in patients who are already profoundly anemic at presentation. Instead, they suggest that a restrictive strategy may be safe for patients with higher baseline hemoglobin, by allowing their levels to fall closer to 7 g/dL before transfusion. Second, they underscore the importance of early risk stratification: distinguishing patients who can safely tolerate lower hemoglobin from those in whom earlier transfusion remains necessary due to comorbidities, hemodynamic instability, or ongoing bleeding.

## 5. Conclusions

This study demonstrates that varying transfusion thresholds do not significantly influence mortality in patients with S-ARDS. Nonetheless, appropriate and timely blood management remains crucial, particularly in patients with severe sepsis or advanced acute lung injury, where transfusion may help stabilize hemodynamics and support tissue oxygenation. These findings suggest that transfusion decisions should be guided not by rigid hemoglobin cutoffs alone, but by a comprehensive assessment of the patient’s clinical status, comorbidities, and disease severity. Future large-scale prospective randomized controlled trials are warranted to better define optimal transfusion strategies across different S-ARDS subgroups, especially those with profound anemia or severe lung injury.

## Author contributions

**Conceptualization:** Kexin Wen, Quansheng Du.

**Investigation:** Quansheng Du.

**Methodology:** Quansheng Du.

**Software:** Quansheng Du.

**Supervision:** Quansheng Du.

**Validation:** Quansheng Du.

**Visualization:** Quansheng Du.

**Writing – original draft:** Kexin Wen.

**Writing – review & editing:** Tianyu Zhao, Ruimin Tan, Xumin Han, Lei Li, Quansheng Du.

## Supplementary Material
