# Supplementary material for: Effects of hemoglobin transfusion thresholds on clinical outcomes in sepsis-related ARDS: A retrospective cohort analysis utilizing the MIMIC-IV database
Source: Medicine (Baltimore). 2025 Sep 26;104(39):e44790. doi: 10.1097/MD.0000000000044790 (PMC13593223; doi:10.1097/MD.0000000000044790)

Supplementary figure 1 Flowchart of included patients.

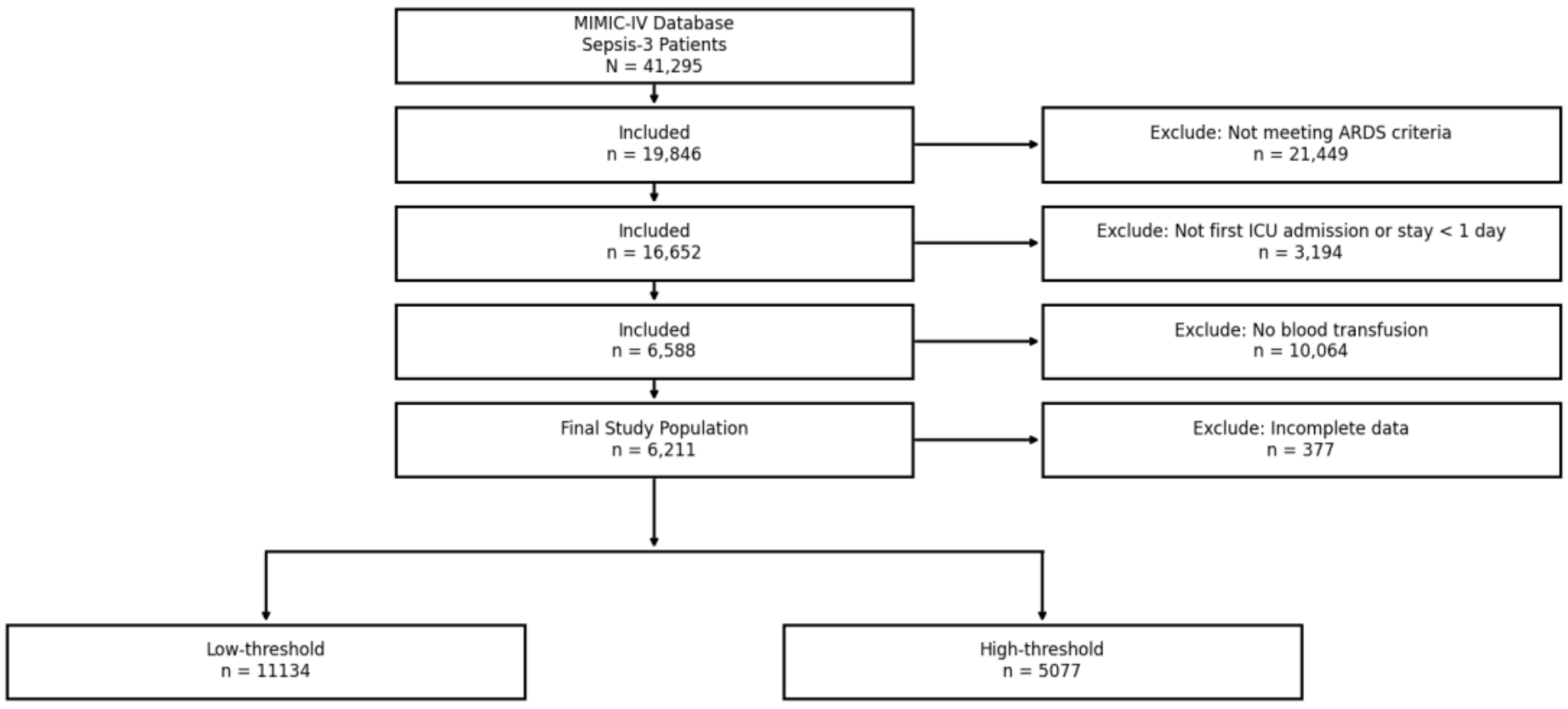

Supplement: Supplementary file 1 [file medi-104-e44790-s001.pdf]
